# The FGFR4-388arg Variant Promotes Lung Cancer Progression by N-Cadherin Induction

**DOI:** 10.1038/s41598-018-20570-3

**Published:** 2018-02-05

**Authors:** Álvaro Quintanal-Villalonga, Laura Ojeda-Márquez, Ángela Marrugal, Patricia Yagüe, Santiago Ponce-Aix, Ana Salinas, Amancio Carnero, Irene Ferrer, Sonia Molina-Pinelo, Luis Paz-Ares

**Affiliations:** 1 Medical Oncology Department, Hospital Universitario Doce de Octubre & Centro Nacional de Investigaciones Oncológicas (CNIO), Madrid, Spain; 2 Instituto de Biomedicina de Sevilla (IBIS) (HUVR, CSIC, Universidad de Sevilla), Sevilla, Spain; 3 Medical School, Universidad Complutense, Madrid, Spain; 4 CIBER de Cáncer, Madrid, Spain

## Abstract

The FGFR4-388Arg variant has been related to poor prognosis in several types of cancer, including lung cancer. The mechanism underlying this association has not been addressed in detail in patients with this pathology. Here, we report that this FGFR4 variant induces MAPK and STAT3 activation and causes pro-oncogenic effects in NSCLC *in vitro* and *in vivo*. This variant induces the expression of EMT-related genes, such as N-cadherin, vimentin, Snail1 and Twist1. Indeed, the induction of N-cadherin protein expression by this variant is essential for its pro-tumorigenic role. The presence of the FGFR4-388Arg variant correlates with higher N-cadherin expression levels in clinical NSCLC samples and with poorer outcome in patients with FGFR expression. These results support the prognostic role of this FGFR variant in lung cancer and show that these effects may be mediated by the induction of N-cadherin expression and an EMT phenotype.

## Introduction

The incidence and mortality of lung cancer are increasing worldwide^1^. Lung cancer is a heterogeneous disease that can be classified into two major histologically distinct groups: small cell lung cancer (SCLC) and non-small cell lung cancer (NSCLC), which accounts for 85% of primary lung carcinomas^2^. Among NSCLC cases, adenocarcinoma (ADC) and squamous cell cancer (SCC) are the most frequent histological subtypes. Currently, lung cancer accounts for the majority of cancer-related deaths; the dismal prognosis of patients with lung cancer is due to the common presentation with advanced stage disease at initial diagnosis and the relative inefficacy of available systemic treatment. Indeed, the expected 5-year survival rate is 18%^3^. Therefore, the identification of relevant diagnostic biomarkers and novel and druggable targets for this disease represents an unmet clinical need.

In recent years, many molecular alterations with a significant role in tumor pathogenesis and the outcome of cancer patients have been identified. Many such alterations have involved tyrosine kinase receptors^4^, which have proven therapeutic potential in a variety of cancer types. One such tyrosine kinase receptor is FGFR4, which has been associated with prognosis in several types of cancer, including lung cancer^5–7^. Several molecular alterations of FGFR4 leading to different gene variants have been identified^8^. One of these variants, FGFR4–388Arg (rs351855 at the genotype level), harbors an amino acid substitution of an arginine for a glycine at codon 388. This FGFR4 variant correlates with disease progression and poorer prognosis in colon, prostate, head and neck, breast, and soft tissue tumors, among many others^9–15^. Nonetheless, the effects of this FGFR4 variant in NSCLC patient prognosis seem to be controversial. In a study involving Asian NSCLC patients, the FGFR4-388Arg variant correlated with poorer outcome in patients with lymph node involvement^16^. However, contradictory results have been reported in other studies. In a work involving advanced NSCLC Asian patients, the FGFR4-388Arg variant correlated with better outcome^17^, and in another study involving Caucasian NSCLC patients, no association between this FGFR4 variant and outcome was found^18^. In some retrospective studies of lung cancer cohorts analyzing each histological subtype independently, the FGFR4-388Arg variant was linked to lymph node involvement and poorer overall survival (OS) in ADC patients^13,16,18,19^. For SCC patients, however, association of this variant with prognosis has been described only in lymph node-involved patients^18–20^.

The FGFR4-388Arg variant has been reported to promote the activation of pathways related to cancer, such as the STAT3 signaling pathway in murine breast and lung cancer models^21^. In addition, overexpression of FGFR4-388Arg was reported to induce MAPK activation and promote proliferation and invasion in *in vitro* models of prostate cancer^22^. Furthermore, this FGFR4 variant has been related to epithelial-to-mesenchymal transition (EMT) in prostate cancer cell lines^23^. In this work, we aimed to study the effect of the FGFR4-388Arg variant on lung cancer oncogenic behavior, its relationship with EMT in this setting, and its impact on patient prognosis.

## Results

### The FGFR4-388Arg variant shows increased tumorigenic potential *in vitro* and *in vivo*

**Figure 1:**
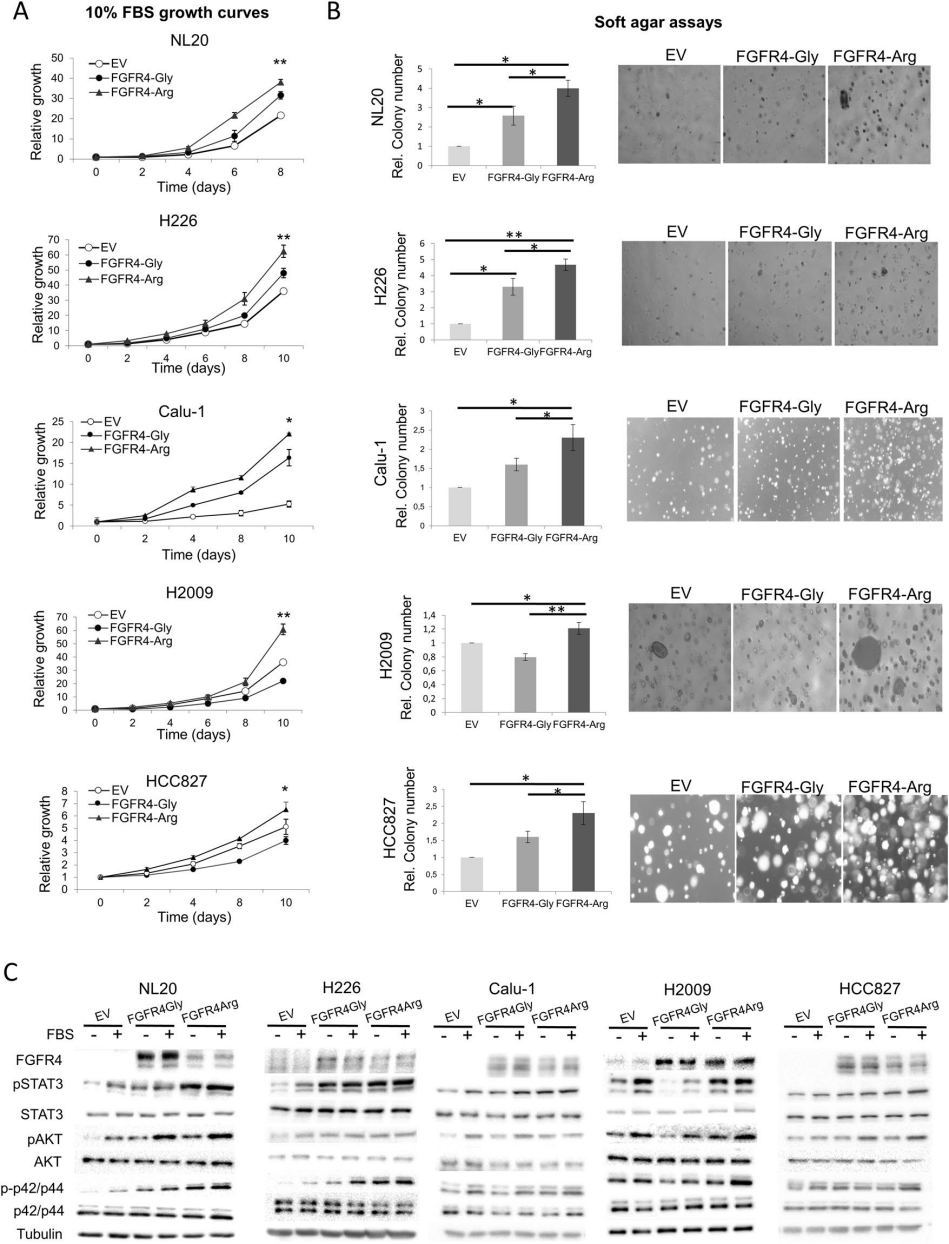
Effect of the overexpression of the 388Gly and 388Arg variants of FGFR4 in the tumorigenic abilities of the inmortalized NL20, the lung SCC H226 and Calu-1, and the H2009 and HCC827 lung ADC cell lines. (**A**) 10% FBS growth curves. (**B**) soft agar assays showing the relative colony number quantification (left) and representative images (right). (**C**) Western blot determination of activation of cancer-related signalling pathways of 388Gly and 388Arg FGFR4-overexpressing cell lines. In soft agar assay, colony number representation is shown. All values were normalized to empty vector control for each replicate of the experiment, and the mean and standard deviation of every normalized replicate are represented. For western blotting, cells were serum starved for 5 hours and then the protein extraction was made. For the serum stimulated conditions, after serum starvation cells were stimulated with serum-containing complete medium for fifteen minutes before protein extraction. p-Values are represented as asterisks (*p < 0,05; **p < 0,01; ***p < 0,001). EV = Empty Vector control, FGFR4-Gly = FGFR4-388Gly overexpressing, FGFR4-Arg = FGFR4-388Arg overexpressing, FBS = Fetal Bovine Serum.

To ascertain the impact of the FGFR4-388Arg variant on the tumorigenic behavior of lung cell lines, we overexpressed the FGFR4-388Gly and FGFR4-388Arg alleles in three lung cell lines that lacked endogenous FGFR4 expression and performed surrogate assays of tumorigenic activity. We selected cell lines with different genetic backgrounds (Supplementary Table S1): one immortalized cell line (NL20), two SCC cell lines (H226 and Calu-1) and two ADC cell lines (H2009 and HCC827). In the NL20, H226, Calu-1 and HCC827 cell lines, ectopic FGFR4-388Gly expression increased cell growth, and clonogenicity and soft agar colony formation compared to the empty vector control, and FGFR4-388Arg elicited a greater increase in these tumorigenic activities (Fig. 1A,B and Supplementary Figure S1). We then determined the activation status of downstream FGFR cancer-related signaling pathways (specifically, the STAT3, AKT and MAPK pathways) in these cell lines; Generally, FGFR4-388Arg overexpression increased pSTAT3 and p-42/p44 levels compared to their respective empty vector control and FGFR4-388Gly overexpressing cell lines. However, AKT signaling was similarly activated in the cell lines overexpressing either FGFR4 variant (Fig. 1C). When we analyzed the effects of overexpressing each variant allele in the lung ADC H2009 cell line, we found that cell growth was slightly reduced by FGFR4-388Gly overexpression but was increased by FGFR4-388Arg overexpression compared to the empty vector control (Fig. 1A). Equivalent results were obtained in the clonogenicity and soft agar assays: in H2009 cells, the empty vector produced more colonies than FGFR4-388Gly but fewer colonies than FGFR4-388Arg (Fig. 1B and Supplementary Figures S1). When the activation of FGFR-related signaling pathways was assessed by western blot, the activation of STAT3, AKT and ERK was decreased in FGFR4-388Gly-overexpressing H2009 cells but increased in FGFR4-388Arg-overexpressing H2009 cells compared to those expressing the empty vector control (Fig. 1C). These results show that although FGFR4-388Gly seems to exert differential effects on tumorigenesis in the cell lines tested, FGFR4-388Arg has a consistent pro-oncogenic role in different lung cancer cell lines. Furthermore, these tumorigenic effects correlate with greater activation of STAT3 and MAPK signaling.

**Figure 2:**
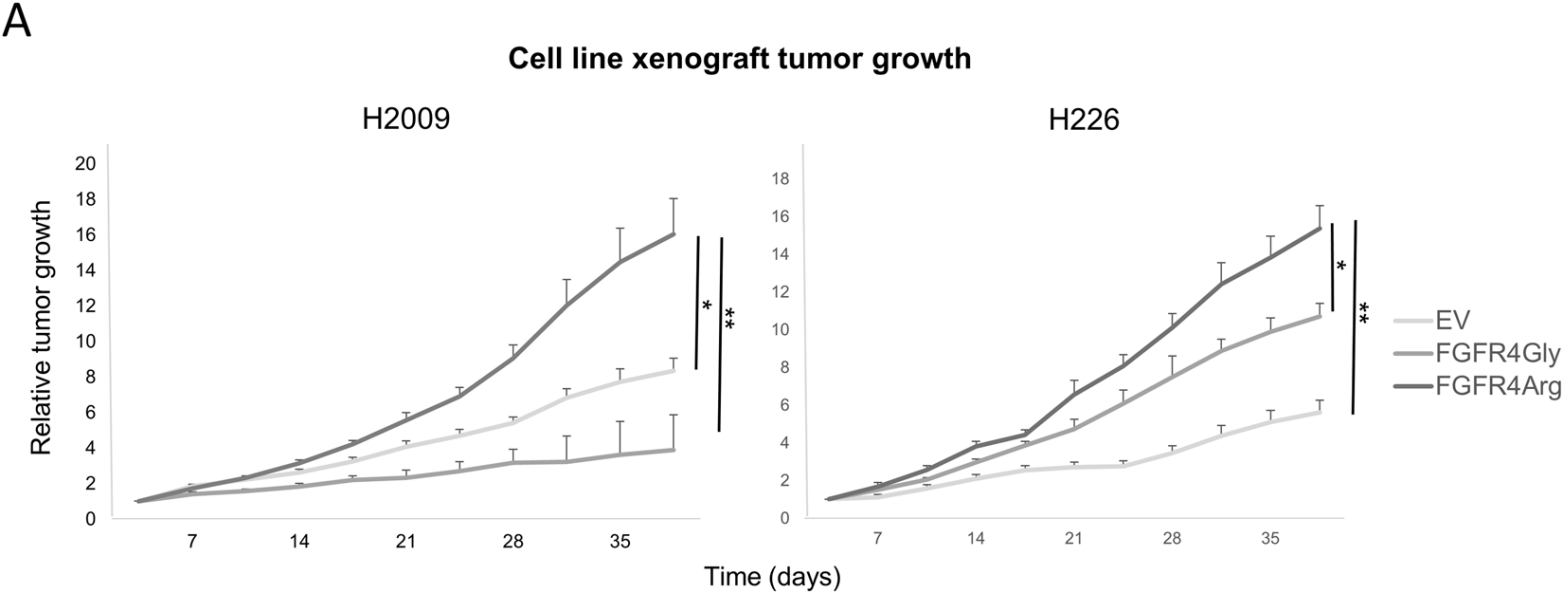
*In vivo* effects of the overexpression of the 388Gly and 388Arg variants in the H2009 ADC and H226 SCC cell lines. Relative tumor growth representation of the xenografts generated in immunodeprived nude mice of the reported cell lines. p-values are represented as asterisks (*p < 0,05; **p < 0,01; ***p < 0,001). EV = Empty Vector control, FGFR4-Gly = FGFR4-388Gly overexpressing, FGFR4-Arg = FGFR4-388Arg overexpressing.

To test the *in vivo* tumorigenic effects of the FGFR4-388Arg variant, we xenografted into nude mice the adenocarcinoma cell line H2009 and the squamous cell carcinoma cell line H226 overexpressing either FGFR4 variant. According to the *in vitro* results, we reported that FGFR4-388Gly overexpression in these xenografts caused decreased tumor growth in the H2009 cell line, and increased tumor growth in the H226 cell line, as compared to their respective control cell lines. However, in both cases, FGFR4-388Arg overexpression caused higher tumor growth, as compared to the rest of conditions (Fig. 2).

### FGFR4-388Arg variant overexpression induces an epithelial-to-mesenchymal transition phenotype in lung cancer cell lines

**Figure 3:**
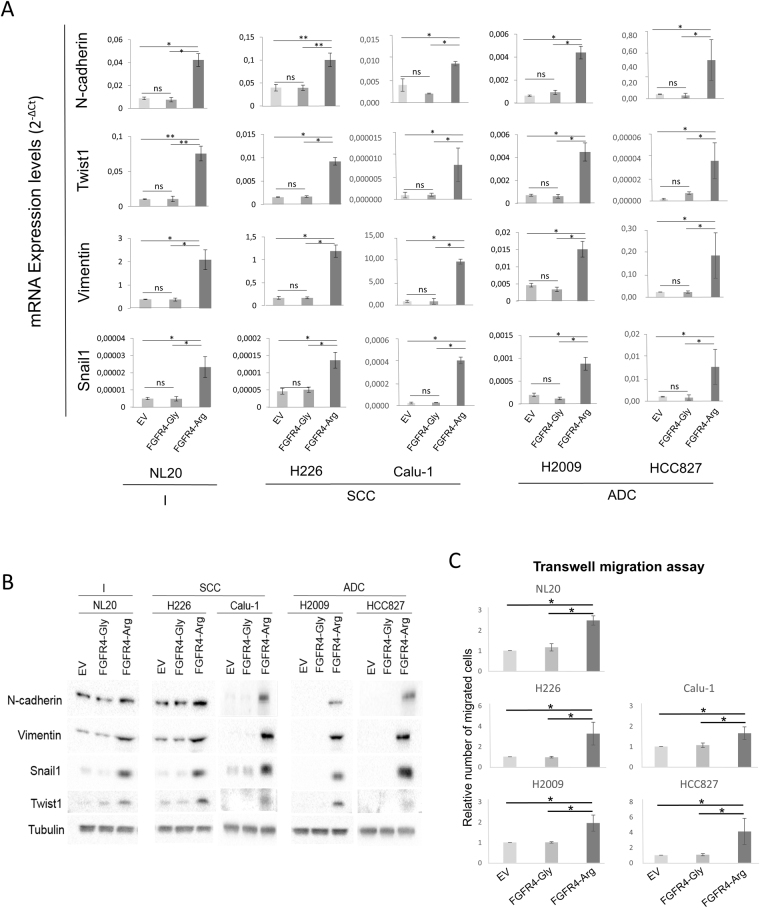
Overexpression of the 388Arg variant of FGFR4 induces the expression of EMT markers in lung cell lines. The mRNA (**A**) and protein (**B**) expression of four EMT markers (N-cadherin, vimentin, Twist1 and Snail1) was performed in the control empty vector (EV), FGFR4-388Gly-overexpressing (FGFR4-Gly) and FGFR4-388Arg-overexpressing (FGFR4-Arg) lung immortalized NL20 cell line and lung SCC H226 and Calu1, and ADC H2009 and HCC827 cell lines. (**C**) Relative number of migrated cells of the previously mentioned cell lines. The mRNA measurements were performed in three independent experiments and mean expression values, represented as 2^−ΔCt^ with their respective standard deviation, are represented. For western blots, a representative blot is shown. Migration assays were performed in three independent experiments. All values were normalized to empty vector control for each replicate of the experiment and the mean and standard deviation of every normalized replicate are represented. p-values are represented as asterisks (*p < 0,05; **p < 0,01; ***p < 0,001, ns = non significant). EV = Empty Vector control, FGFR4-Gly = FGFR4-388Gly overexpressing, FGFR4-Arg = FGFR4-388Arg overexpressing.

It has been reported in other tumor models that silencing FGFR4-388Arg induces a mesenchymal-to-epithelial transition, suggesting a role for FGFR4-388Arg in EMT^22,24^. To explore whether FGFR4-388Arg overexpression induces EMT in lung cell lines, we measured the mRNA and protein levels of several EMT markers (N-cadherin, Twist1, vimentin, and Snail1) in the FGFR4-388Gly- and FGFR4-388Arg-overexpressing cell lines. FGFR4-388Gly overexpression in the five cell lines did not alter the mRNA levels of any of the studied EMT markers compared to their respective empty vector-expressing cell line. However, FGFR4-388Arg overexpression increased the mRNA and protein levels of N-cadherin, vimentin, Snail1 and Twist1 (Fig. 3A,B). In addition, we tested the migration ability of these cell lines as a functional assay for EMT induction. While FGFR4-388Gly overexpression did not increase migration, as compared to the respective control cell lines, the number of migrating cells was higher in the condition of FGFR4-388Arg overexpression in all cell lines under study (Fig. 3C and Supplementary Figure S2). These results indicate that FGFR4-388Arg has a role in inducing an EMT phenotype in lung cancer.

### The pro-oncogenic role of FGFR4-388Arg depends on N-cadherin expression

**Figure 4:**
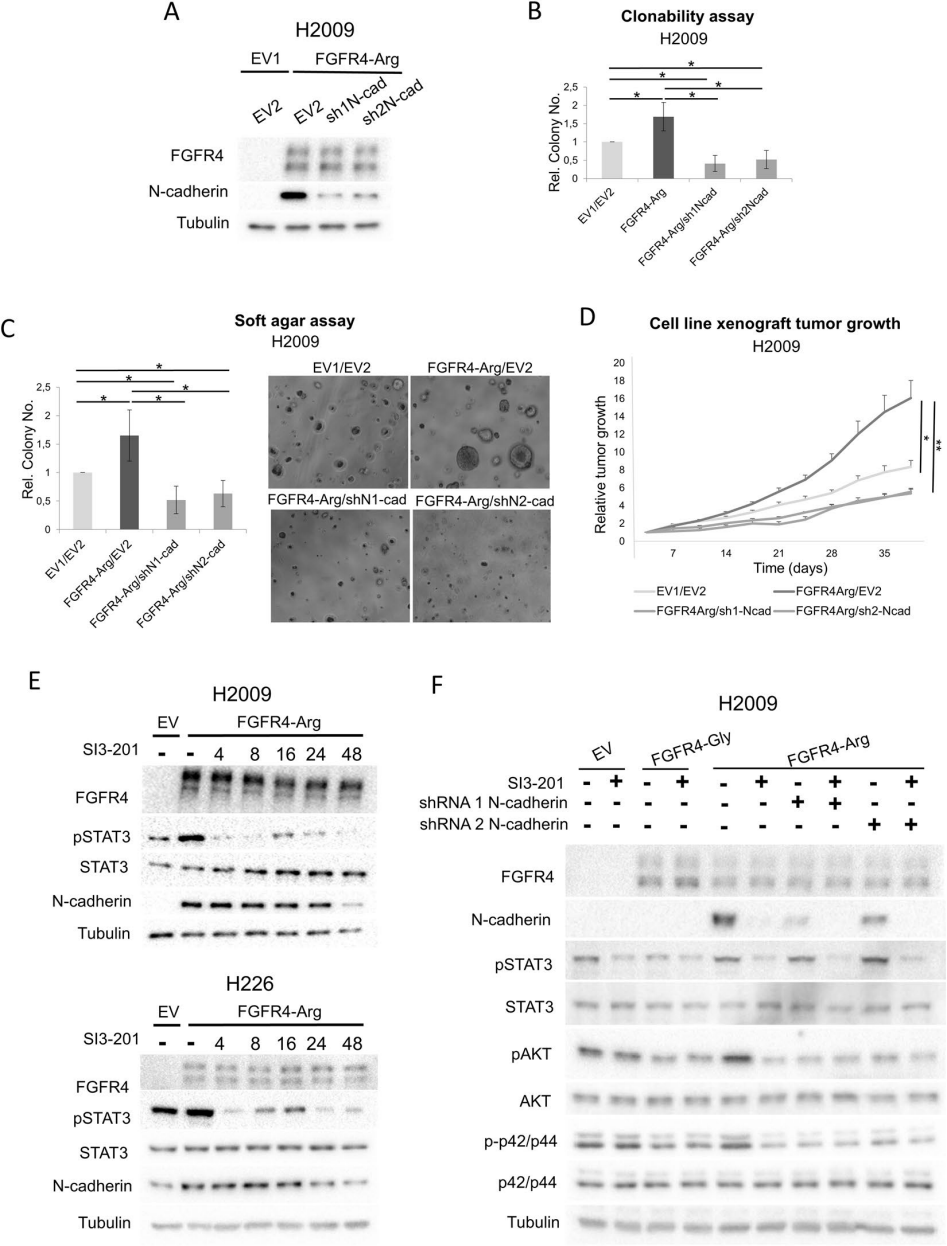
N-cadherin is involved in the pro-oncogenic role of Arg388 FGFR4 overexpression. (**A**) N-cadherin silencing in the FGFR4-388Arg-overexpressing H2009 cell line using a shRNA approach. Clonability (**B**) and soft agar assays (**C**) of the FGFR4-388Arg-overexpressing, N-cadherin silenced H2009 cell line. (**D**) Relative tumor growth of the xenograft in immunodeprived nude mice of these cell lines. (**E**) Western blot showing the STAT3 activation suspension by the STAT3 inhibitor SI3-201 along the time, which is accompanied by N-cadherin protein levels reduction, in the FGFR4-388Arg-overexpressing H2009 and H226 cell lines. SI3-201 exposure in hours is indicated in the figure. (**F**) Western blot showing the effect of STAT3 inhibition and N-cadherin shRNA silencing in several cancer-related downstream signaling pathways in the EV, FGFR4-388Gly and -388Arg-overexpressing H2009 cell line. In soft agar and clonability assays, colony number representation is shown. All values were normalized to empty vector control for each replicate of the experiment and the mean and standard deviation of every normalized replicate are represented. For western blots, a representative blot is shown. EV = Empty Vector control, FGFR4-Gly = FGFR4-388Gly overexpressing, FGFR4-Arg = FGFR4-388Arg overexpressing. p-Values are represented as asterisks (*p < 0,05; **p < 0,01; ***p < 0,001). EV1 = Empty Vector 1, EV2 = Empty vector 2, FGFR4-Gly = FGFR4-388Gly overexpressing, FGFR4-Arg = FGFR4-388 FGFR4 overexpressing, shN1-cad = N-cadherin shRNA 1 silenced, shN2-cad = N-cadherin shRNA 2 silenced.

As N-cadherin expression has been linked to oncogenicity^25–27^, we hypothesized that the overexpression of this adhesion molecule may underlie the pro-tumorigenic potential of FGFR4-388Arg expression. To evaluate this hypothesis, we silenced N-cadherin expression in the FGFR4-388Arg-overexpressing H2009 cell line (Fig. 4A) and performed surrogate assays to ascertain the role of N-cadherin in the induced pro-oncogenic properties. N-cadherin downregulation decreased the clonogenicity and soft agar colony formation compared to the FGFR4-388Arg-overexpressing cell line and the empty vector control cell line (Fig. 4B,C), and the same effects were reported *in vivo* in tumor growth of xenografts in inmunodeprived mice with these cell lines (Fig. 4D). These results support the dependence of FGFR4-388Arg on N-cadherin to promote oncogenic activity.

### STAT3 inhibition reduces the FGFR4-388Arg-induced overexpression of N-cadherin *in vitro*

As STAT3 activation has been related to EMT, we examined whether STAT3 over-activation is involved in the induction of N-cadherin expression, which our data showed to be relevant in the pro-oncogenic effects of FGFR4-388Arg overexpression. We abrogated STAT3 activation in the FGFR4-388Arg-overexpressing H2009 and H226 cell lines with a selective STAT3 inhibitor, SI3-201, and measured N-cadherin protein levels at different time points. Inhibition of pSTAT3 levels was confirmed after 4 hours of treatment, and a reduction in N-cadherin protein levels was reported after 16–24 hours of treatment, with a dramatic reduction at 48 hours (Fig. 4E). These results indicate that STAT3 signaling mediates the induction of N-cadherin expression.

To gain mechanistic insight into these phenomena, we assessed the activation of the STAT3, AKT and MAPK signaling pathways under different conditions in the H2009 lung ADC cell line (Fig. 4F). FGFR4-388Arg overexpression in H2009 cells activated the STAT3, AKT and MAPK signaling pathways compared to control and FGFR4-388Gly-overexpressing cells, as shown before. Treatment with SI3-201, the STAT3 inhibitor, abrogated STAT3 activation in all cell lines. This inhibition did not influence the activation of AKT or MAPK in control cells or FGFR4-388Gly-overexpressing cells. However, STAT3 inhibition reduced AKT and MAPK activation in parallel with N-cadherin expression in FGFR4-388Arg-overexpressing H2009 cells. Furthermore, N-cadherin silencing in the FGFR4-388Arg-overexpressing cell line dramatically inhibited AKT and MAPK activation (Fig. 4F). These results show that FGFR4-388Arg elicits STAT3 activation, which induces N-cadherin expression, and that N-cadherin is likely necessary for the consequent activation of AKT and MAPK.

### FGFR4-388Arg expression correlates with higher N-cadherin expression in human tumors

**Figure 5:**
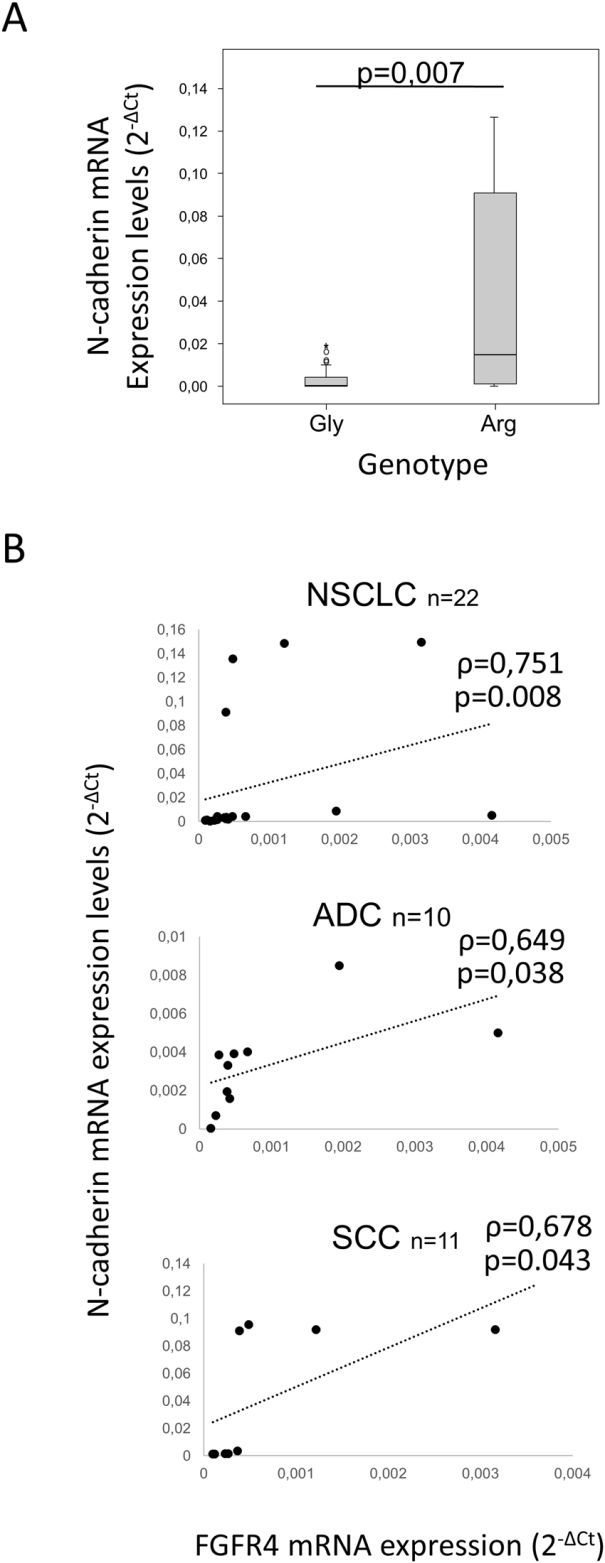
Arg388 FGFR4 mRNA expression correlates with N-cadherin mRNA expression. (**A**) N-cadherin mRNA expression levels according to Arg388 FGFR4 mRNA expression. (**B**) Bivariate correlation analysis of Arg388 FGFR4 and N-cadherin mRNA expression levels in the whole NSCLC cohort and in the ADC and SCC patient subsets. Gly = FGFR4-388Gly, Arg = FGFR4-388Arg.

We determined the FGFR4 Gly388Arg genotype and the mRNA expression of FGFR4 and N-cadherin in a cohort of 65 NSCLC tumors using DNA and RNA extracted from FFPE tumor samples. The characteristics of this patient cohort are shown in Supplementary Table S2. Patients were divided in two groups according to FGFR4 Gly388Arg genotype: homozygous for FGFR4-388Gly (N = 43) and heterozygous for the FGFR4-388Arg variant (N = 22). No homozygous FGFR4-388Arg patients were detected in our cohort. No significant differences between clinicopathological characteristics were found between both patient subgroups. In agreement with our *in vitro* results, we found an association between the FGFR4-388Arg genotype and higher N-cadherin expression in these samples (Fig. 5A). Furthermore, when we selected patients with the 388Arg variant, there was a correlation between FGFR4 and N-cadherin mRNA expression in the whole cohort (Spearman’s coefficient = 0.751, p = 0.008), the ADC group (Spearman’s coefficient = 0.649, p = 0.038) and the SCC group (Spearman’s coefficient = 0.678, p = 0.043) (Fig. 5B). Such correlation was not observed in the FGFR4-388 Gly expressing variant subgroup (data not shown).

### The FGFR4-388Arg variant correlates with poorer survival in NSCLC patients with high FGFR4 expression

**Figure 6:**
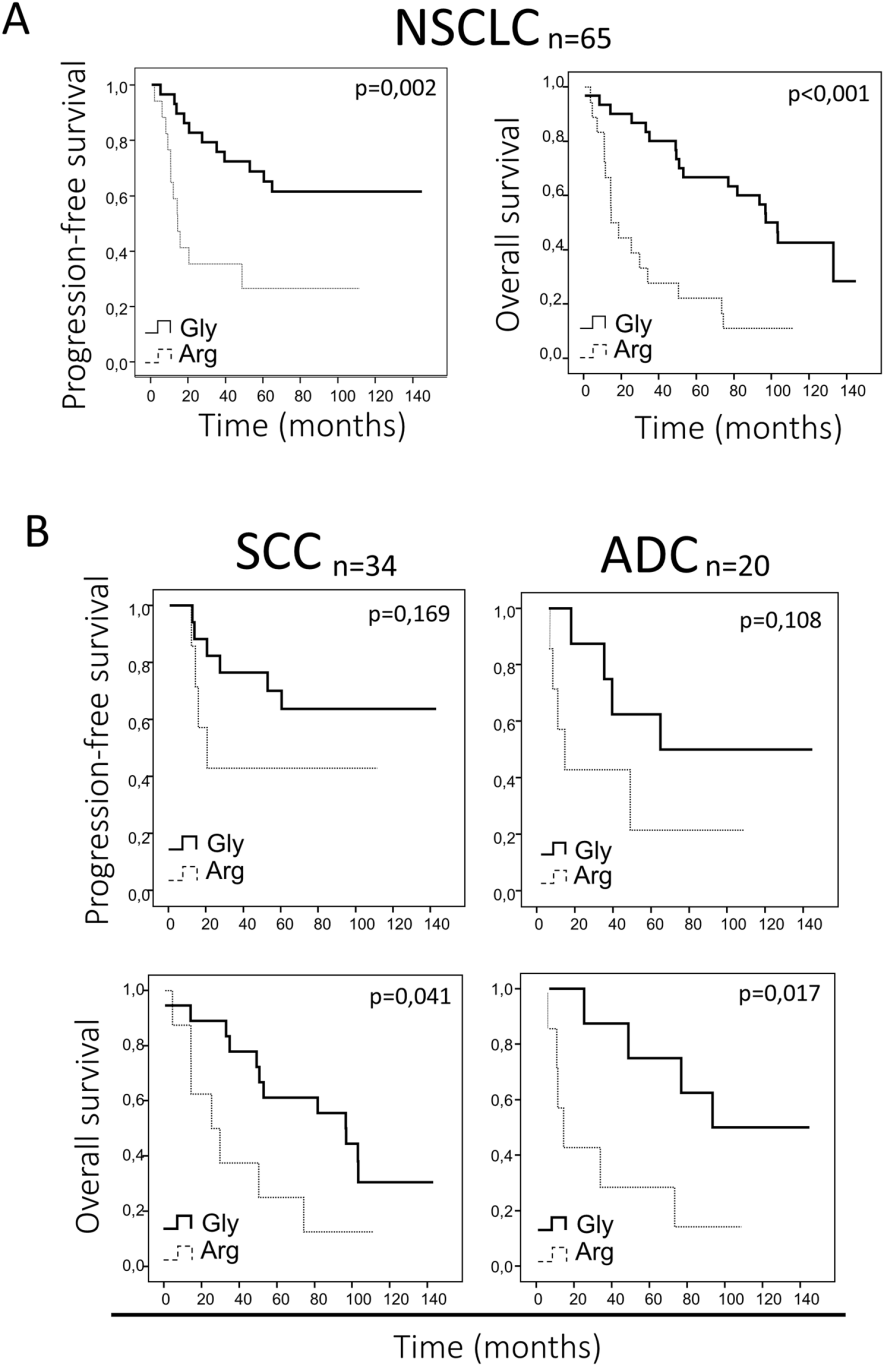
Correlation of FGFR4 variant and prognosis in high FGFR4 mRNA expressing NSCLC patients. (**A**) Overall and progression-free survival curves for high FGFR4 mRNA expressing patients, according to the FGFR4 variant in the whole NSCLC cohort. (**B**) Overall and progression-free survival analysis of SCC and ADC patient subsets depending on the FGFR4 variant, taking into account exclusively the groups with high FGFR4 mRNA expression. Gly = FGFR4-388Gly, Arg = FGFR4-388Arg.

Next, the FGFR4 Gly388Arg genotype was correlated with prognosis in patients with high FGFR4 mRNA expression. We selected the 75% of patients with the highest expression in our cohort (Quartile 2-Quartile 4). We found that the FGFR4-388Arg variant correlated with poorer PFS and OS (p < 0.001 and p = 0.002, respectively; Fig. 6A). This prognostic effect for OS was independent of histology (ADC, p = 0.041; SCC, p = 0.017; Fig. 6B); the same trend was observed for PFS, but statistical significance was not reached (Fig. 6B). However, when we performed multivariate analysis in these patients, we reported that the FGFR4-388Arg variant was not an independent prognostic factor (data not shown).

## Discussion

In this work, we report the pro-oncogenic role of the FGFR4-388Arg variant in lung cancer; this variant correlates with greater STAT3 and MAPK activation and higher expression of EMT markers *in vitro*. This pro-tumorigenic role is mediated by the induction of N-cadherin expression, which requires STAT3 overactivation. At the clinical level, we report a correlation between the FGFR4-388Arg variant and higher N-cadherin expression in lung tumors, as well as an association of this FGFR4 variant with poorer outcomes for patients with NSCLC, regardless of histology.

The FGFR4-388Arg variant correlates with disease progression and poorer prognosis in several types of cancer^9–15^. However, the relationship between this variant and prognosis in NSCLC remains unclear^16–18^ and the effects of this FGFR4 variant in lung pro-tumorigenicity have not been addressed. We report that in the lung cell lines tested, overexpression of FGFR4-388Arg elicited greater pro-tumorigenic effects *in vitro* and *in vivo* than overexpression of the FGFR4-388Gly variant or the empty vector control, independent of histological origin. At a molecular level, this increase in tumorigenic behavior induced by FGFR4-388Arg was accompanied by greater STAT3 and MAPK activation. The substitution of the conserved glycine 388 residue with an arginine residue in FGFR4 exposes a STAT3 binding site, which enhances STAT3 tyrosine phosphorylation^21^. This may explain the overactivation of STAT3 reported in the FGFR4-388Arg-overexpressing cell lines. FGFR4-388Gly overexpression exerted pro-oncogenic effects in most of the cell lines under study compared to the empty vector control cell lines, but unexpectedly caused anti-tumorigenic effects in one of the ADC cell lines. Another member of the FGFR family, FGFR3, has been suggested to exert anti-oncogenic effects in certain contexts in pancreatic cancer cell lines^28^, and FGFR4 could behave similarly under specific molecular backgrounds. However, the potential anti-tumorigenic role of FGFR4-388Gly should be reproduced in additional lung cancer cell lines and further studied in order to be confirmed.

It has been reported that FGFR4 silencing in FGFR4-388Arg-expressing prostate cancer cell lines leads to the induction of E-cadherin expression and to decreased N-cadherin expression^29^, suggesting that FGFR4-388Arg may be related to EMT. To ascertain if FGFR4-388Arg induces an EMT phenotype in lung cell lines upon overexpression, the expression of several EMT markers (N-cadherin, Twist1, vimentin, and Snail1) was assessed by RT-qPCR and western blot. We confirmed that this FGFR4 variant is potentially involved in inducing EMT, as every EMT marker tested showed increased expression exclusively in the cell lines overexpressing FGFR4-388Arg, which furthermore showed increased migration ability. N-cadherin has been extensively implicated in cancer progression^25,30,31^ and in anti-apoptotic mechanisms in NSCLC cell lines^26^. Thus, we hypothesized that N-cadherin could be mediating the pro-oncogenic effects observed in these cell lines, so we downregulated N-cadherin expression in the FGFR4-388Arg-overexpressing H2009 lung ADC cell line, and determined the effects on tumorigenesis by performing surrogate assays. Our data showed that in this cell line, N-cadherin silencing reduced MAPK and AKT signaling, and reversed the pro-oncogenic effects of FGFR4-388Arg overexpression *in vitro* and *in vivo*, supporting the hypothesis that N-cadherin upregulation in response to FGFR4-388Arg overexpression is involved in the increased pro-oncogenic activity caused by this FGFR4 variant. In accordance with our results, pro-tumorigenic effects accompanied by MAPK overactivation have been reported in the context of N-cadherin and FGFR co-expression^32^, and AKT activation by this adhesion molecule has been reported in several contexts^33,34^, which may explain the increase in oncogenic signaling and pro-tumorigenicity upon N-cadherin induction by the FGFR4-388Arg variant.

Next, we wanted to elucidate the mechanism by which FGFR4-388Arg overexpression is pro-tumorigenic. Overexpression of this FGFR4 variant caused STAT3 overactivation. Constitutive STAT3 signaling has been related to EMT; this signaling pathway has been reported to be involved in downregulating epithelial phenotype-related genes and inducing a mesenchymal phenotype^35–37^. In hepatocellular carcinoma, STAT3 binds to the Twist promoter and induces its expression, thus triggering EMT and N-cadherin expression^37^. Considering these data, we aimed to determine if STAT3 overactivation caused by FGFR4-388Arg overexpression is linked to N-cadherin upregulation. Therefore, we analyzed the effects of a STAT3 selective inhibitor on N-cadherin protein levels in FGFR4-388Arg-overexpressing cell lines. STAT3 inhibition decreased N-cadherin protein expression, supporting the idea that constitutive activation of the STAT3 signaling pathway is involved in upregulating N-cadherin. In the H2009 cell line, the STAT3 inhibitor abrogated STAT3 activation in both FGFR4-388Gly- and FGFR4-388Arg-overexpressing conditions but, interestingly, decreased AKT and MAPK activation, accompanied by N-cadherin downregulation, in only the FGFR4-388Arg-overexpressing cell line. Furthermore, N-cadherin silencing in the FGFR4-388Arg-overexpressing cell line decreased the activation of the AKT and MAPK signaling pathways. These results suggest that N-cadherin upregulation is linked to MAPK overactivation in response to FGFR4-388Arg overexpression, independent of STAT3 activation. All these data support a model wherein FGFR4-388Arg increases STAT3 activation, which consequently upregulates N-cadherin expression. Then, N-cadherin upregulation increases AKT and MAPK signaling, which may be ultimately responsible for the pro-oncogenic characteristics reported in these cell lines.

We provide clinical evidence that FGFR4-388Arg is associated with higher N-cadherin mRNA expression in a cohort of NSCLC patients. We found a correlation between N-cadherin and FGFR4 mRNA expression in patients with the FGFR4-388Arg variant. These data are in accordance with our *in vitro* results, which showed that FGFR4-388Arg overexpression increased N-cadherin expression levels.

In previous retrospective studies, the association of the FGFR4-388Arg genotype to prognosis in lung cancer patients has remained controversial^13,16–19^. Some of them independently analyzing NSCLC histological subtypes agreed on a potential prognostic role of this variant in ADC patients^13,19^, and in lymph node-affected squamous cell carcinoma patients^19,20^. In our patient cohort that included patients with ADC, SCC and a few other histologic types of lung cancer, we found that the FGFR4-388Arg variant correlated with poor OS and PFS in patients with high FGFR4 mRNA expression. Furthermore, when we independently analyzed the SCC or ADC patients, FGFR4-388Arg-expressing tumors correlated with worse OS and showed a correlation trend with PFS in both patient subsets, suggesting that FGFR4-388Arg expression may have a prognostic role in both histologic types. For all the prognosis analyses presented herein, we considered only patients with high FGFR4 mRNA expression; we discarded patients with low or absent FGFR4 gene expression. This criterion was formulated because expression of the variant is required for it to exert any effects. In contrast, previously published studies in the context of NSCLC considered only the FGFR4 allelic variant, not gene expression, in the tumor. This may explain why these previous results were not reproducible in the different cohorts. However, multivariate analysis of survival in these patients did not support the independent prognostic capacity of the FGFR4 variant, which may be explained by the relatively low number of patients included in our analysis. Our results should be further confirmed in independent cohorts including a higher number of patients, and considering not only the FGFR4 variant present in the tumor, but also its expression level.

In summary, we have shown here that the FGFR4-388Arg variant has an impact on the tumorigenic behavior of lung cancer cell lines by inducing an EMT expression profile through increased N-cadherin expression and overactivation of MAPK and AKT signaling. Furthermore, we report clinical evidence that this variant has a potential prognostic role in lung cancer patients with tumors that express high levels of FGFR4. We provide *in vitro*, *in vivo*, mechanistic and clinical data supporting the potential pro-oncogenic and prognostic role of the FGFR4-388Arg variant in NSCLC.

## Methods

### Cell lines and transfections

The NL20, H226, Calu-1, HCC827 and H2009 cell lines were purchased from American Type Culture Collection (ATCC) at the beginning of this study and were cultured according to instructions from ATCC. Cells were authenticated and regularly checked for mycoplasma contamination.

Cell lines were transfected with overexpression plasmids (pCMV6, PS100001, Origene) carrying the FGFR4-388Gly (RG204230, Origene) or the FGFR4-388Arg (RC402575, Origene) alleles using TransIT-X2 transfection reagent (Mirus). The empty vector was transfected into the cell lines as a negative control. For N-cadherin silencing, shRNAs in the pB-RS plasmid were purchased from Origene (HC138304). Two different shRNAs successfully downregulating N-cadherin expression were used for further experiments to avoid off-target effects. The appropriate antibiotic (1 mg/mL G418 or 1-4 µg/mL blasticidin) was used to select positive clones, which were pooled in a monolayer culture to generate stable cell lines.

### Immunoblotting

Protein was extracted from cell lines using RIPA buffer (Sigma) supplemented with a protease inhibitor cocktail (cOmplete Mini EDTA-free, Roche) and a phosphatase inhibitor cocktail (PhosSTOP EASYpack, Roche). A standard western blot protocol was utilized with a miniProtean electrophoretic system (BioRad) and a semi-dry electrotransfer system (BioRad). Primary antibodies against FGFR4 (#8562, Cell Signaling), AKT (#9272, Cell Signaling), pAKT (#9271, Cell Signaling), ERK1/2 (#9102, Cell Signaling), pERK1/2 (#9101, Cell Signaling), STAT3 (#9139, Cell Signaling), pSTAT3 (#9145, Cell Signaling), N-cadherin (#3195, Cell Signaling), Vimentin (#5741, Cell Signaling), Twist (#46702 S, Cell Signaling), Snail (#3879 S, Cell Signaling) and α-tubulin (#T9026, Sigma) were used. α-Tubulin protein expression was used as the loading control. Horseradish peroxidase-conjugated secondary antibodies were used for chemiluminescence-based detection of protein expression in the ChemiDoc detection system (BioRad). No grouping of gels/blots cropped from different parts of the same gel, or from different gels, fields, or exposures was performed.

### Cell line stimulation

Cell lines were stimulated with fetal bovine serum (FBS) in culture media at a routine concentration: 10% FBS for H226 and H2009 cells and 4% for NL20 cells. Cells were incubated in FBS-free medium for five hours to achieve the basal phosphorylation state. Then, the cells were stimulated by the addition of growth medium for 15 minutes, and protein extracts were obtained as indicated above.

### STAT3 inhibition

STAT3 inhibition experiments were performed using the SI3-201 inhibitor (Selleckchem). The IC20 (concentration at which growth is reduced at 20% in 48 hours) was calculated^38^ and applied to the cell line under assessment in complete growth medium. Culture medium with the inhibitor was renewed after 24 hours. Protein was extracted at 4, 8, 16, 24 and 48 hours.

### Cell line RNA extraction and analysis

RNA was extracted from cell lines using Trizol Reagent (Life Technologies) and then reverse transcribed with the TaqMan Reverse Transcription Kit (Life Technologies). Gene expression analysis was performed using TaqMan probes from Life Technologies: Hs00983056_m1 FAM (N-cadherin), Hs00195591_m1 (SNAI1), Hs01675818_s1 (TWIST1), Hs00185584_m1 (VIM) and Hs99999905_m1 FAM (GAPDH). GAPDH was used as a reference gene to normalize expression data.

### Clonogenicity assays

A limited number of cells, typically 3000, corresponding to the clonogenic density was seeded in 10-cm cell culture plates. Each condition was seeded in triplicate per assay, and each assay was repeated a minimum of three times. Medium was renewed once a week during the assay, and after two or three weeks, depending on the cell line, the plates were fixed with 0.5% glutaraldehyde for 20-30 minutes. Then, the cells were stained with a 1% crystal violet solution in water. The plates were washed and the colonies counted.

### Growth curves

A total of 3500 cells per well were seeded in 12-well culture plates (Nunc) in complete growth medium. Three replicates per condition were assayed in each growth curve, and a minimum of three replicate growth curves were generated for each experiment. The first point of the curve (day 0) was fixed with a 0.5% glutaraldehyde solution 24 hours after seeding. In the 0.5% FBS growth curves, cells were washed twice with PBS, and medium containing 0.5% FBS was then added before the day 0 plate was fixed. Every two days, a point on the curve was fixed and stored in PBS at 4 °C until the last point of the curve was fixed. Cells were then stained with a 1% crystal violet solution and washed. The crystal violet that attached to the cells was dissolved in a 20% acetic acid/water solution, and the absorbance of this solution at 595 nm was measured. The intensity of the absorbance correlates with the quantity of cells, and the data were normalized to the absorbance on day 0. The normalized absorbance over time is presented.

### Soft agar assays

A total of 100,000 cells per well in 0.35% agarose growth medium were seeded in 6-well plates over a base of 0.7% agarose medium. The day after seeding, 3 mL of complete growth medium was added to each well. The media was replaced twice a week until the end of the experiment, at which point the colonies had undergone sufficient growth. Then, photos were taken of each well with microscope (#IX2-SLP, Olympus) with an installed camera (#U-CMAD3, Olympus), and colony number and size were determined.

### Migration assays

Cells were tripsinized, counted, and included in FBS-free medium. 150.000 cells in 1.5 mL of FBS-free medium were added to 8 µM pore size 6-well transwells (#3428. Cultek). Transwells were placed on 6-well plates including 2.5 mL of 10% FBS medium. After 48 hours of incubation, transwell were discarded and migrated cells attached to the well bottom were fixed and stained with a 1% crystal violet solution. The crystal violet that attached to the cells was dissolved in a 20% acetic acid/water solution, and the absorbance of this solution at 595 nm was measured. The intensity of the absorbance correlates with the quantity of migrated cells.

### Mouse xenograft and tumor growth assessment

Cell lines were trypsinized, counted and diluted in PBS. Then, the cell suspension was mixed with Matrigel (1:1), and 150 µL (containing 1 × 10^6^ cells) of the mixture was injected into both flanks of female, 6-week-old athymic nude mice (nu+/nu+). 5 animals were included in each group to reach statistical significance, based on previous experiments in the laboratory, and reviewed and approved by the Animal Protection Comittee. Tumors were measured weekly after implantation, and the mice were sacrificed when the tumor volume exceeded 1000 mm^3^. Then, tumors were harvested and stored.

### Clinical specimens

The present study involved 65 subjects from the Virgen del Rocio University Hospital (Seville, Spain). Patients had undergone surgical resection, and tumor samples were sent to the pathology laboratory for diagnosis and were prepared for storage by formalin fixation and paraffin embedding.

### Ethical issues

Regarding human samples, written informed consent was provided by all the patients. The project was approved by the Research Ethics Committee of the Virgen del Rocío University Hospital, Sevilla, Spain (Approval ID: 2012PI/241).

The procedures involving animals were approved by Animal Protection of the Comunidad Autónoma de Madrid (Approval ID: PROEX134/16).

All experiments were performed in accordance with relevant guidelines and regulations. Biobanking and handling of the human samples followed the BRISQ guidelines^39^. And, for tumor marker prognostic study, the REMARK reporting guidelines were followed^40^.

### Genotyping

DNA was extracted from tissue sections of formalin-fixed, paraffin-embedded (FFPE) samples using the QIAamp DNA Mini Kit (#51306, QIAGEN), and the DNA concentration was measured using a Nanodrop ND-1000 spectrophotometer (Nanodrop Tech). DNA samples were then preamplified using Preamplification Master Mix (#4384266, Applied Biosystems) and the rs351855 TaqMan Genotyping probe (#4351379, Applied Biosystems), following the manufacturer’s instructions, with an 18-cycle preamplification protocol; then, the samples were diluted 1:20. Genotyping was conducted using the 50-cycle genotyping protocol from TaqMan and the rs351855 probe, and the results were analyzed with TaqMan Genotyper software.

### RNA extraction from clinical specimens

RNA was extracted from paraffin-embedded tissue from patients using the RecoverAll Extraction Kit (#AM1975, Life Technologies). RNA samples were then reverse transcribed with the TaqMan Reverse Transcription Kit (Life Technologies). Prior to the analysis of each gene, a preamplification step was performed with the TaqMan Preamp Master Mix Kit (#4384266, Applied Biosystems). Preamplification and gene expression analysis were conducted using TaqMan probes from Life Technologies: Hs01106908_m1 FAM (FGFR4), Hs00983056_m1 FAM (N-cadherin), and Hs99999905_m1 FAM (GAPDH). GAPDH was used as the reference gene to normalize the expression data.

### Statistical analysis

Statistical analysis was performed with the SPSS statistical package (v19, IBM). Differences between experimental conditions were analyzed using Student’s t-test. Spearman’s Rho method was used for bivariate correlation analysis. Kaplan-Meier curves were generated to calculate overall survival (OS) and progression-free survival (PFS), and significant differences were assessed by log rank univariate and proportional hazards regression multivariate analysis. The Chi-Square test was used to assess differences in clinicopathological characteristics between the different patient subgroups.

## Electronic supplementary material

Supplementary table S1, Supplementary table S2, supplementary figure S1 and supplementary figure S2.
