# Supplementary material for: The FGFR4-388arg Variant Promotes Lung Cancer Progression by N-Cadherin Induction
Source: Sci Rep. 2018 Feb 5;8:2394. doi: 10.1038/s41598-018-20570-3 (PMC5799167; doi:10.1038/s41598-018-20570-3)
Supplement: Supplementary file 1 — Supplementary table S1, Supplementary table S2, supplementary figure S1 and supplementary figure S2. [file 41598_2018_20570_MOESM1_ESM.pdf]

Title: THE FGFR4-388ARG VARIANT PROMOTES LUNG CANCER PROGRESSION BY N-CADHERIN INDUCTION

Authors and affiliations

Álvaro Quintanal-Villalonga[1], Laura Ojeda-Márquez[1], Ángela Marrugal[1], Patricia Yagüe[1], Santiago Ponce-Aix[1,4], Ana Salinas[2], Amancio Carnero[2,4], Irene Ferrer[1,4]*, Sonia Molina-Pinelo[2,4*], Luis Paz-Ares[1,3.4]*

1. Medical Oncology Department, Hospital Universitario Doce de Octubre & Centro Nacional de Investigaciones Oncológicas (CNIO), Madrid, Spain.

2. Instituto de Biomedicina de Sevilla (IBIS) (HUVR, CSIC, Universidad de Sevilla), Sevilla, Spain.

3. Medical School, Universidad Complutense, Madrid, Spain.

4. CIBER de Cáncer

*Corresponding authors. These authors contributed equally to this work.

SUPPLEMENTARY INFO

Supplementary Table S1. Description of the driver molecular alterations and FGFR1 and FGFR4 mRNA expression of our lung cell line panel **Figure S1**. Related to **Figure 1**. ADC=Adenocarcinoma, SCC=Squamous Cell Carcinoma, TN="Triple Negative" (referring to the absence of alterations in KRAS, EGFR or ALK), I=Immortalized.

Cell line	Histology	Driver mutation	Reference for driver mutation
H2009	ADC	KRAS p.G12A	COSMIC
HCC827	ADC	EGFR E746-E750 del	(Helfrich, Raben et al. 2006)
Calu-1	SCC	KRAS p.G12C	COSMIC
H520	SCC	TN	COSMIC, (Helfrich, Raben et al. 2006)
H226	SCC	TN	COSMIC, (Helfrich, Raben et al. 2006)
NL20	I	TN	COSMIC

Supplementary Table S2. Characteristics of the resected lung cancer patient cohort.

	Whole cohort	Gly	Arg	p-value
**Subjects, n**	65	43	22	
**Gender**				
**Male**	63 (96.9%)	42 (97.6%)	21 (95.5%)	
**Female**	2 (3.1%)		1 (4.5%)	0.624
		1 (2.4%)		
**Median age, years**	65.0 [54.0-78.0]		64 [54.0-77.0]	0.726
		66.0 [52.0-		
**Smoking status**		79.0]		
**Smoker**	34 (52.3%)		12 (54.5%)	
**Ex-smokers**	29 (44.6%)		9 (40.9%)	
**Never-**	2 (3.1%)	22 (51.1%)	1 (4.9%)	0.832
**smokers**		20 (46.5%)		
**Squamous cell**		1 (2.0%)		
**carcinoma**	34 (52.3%)		11 (50.0%)	
**Adenocarcinoma**	20 (30.7%)		10 (45.4%)	
**Large cell carcinoma**	11 (17%)	23 (53.4%)	1 (4.6%)	0.068
		10 (23.3%)		
**Stage I-II**	34 (52.3%)	10 (23.3%)	11 (50.0%)	
**Stage III**	31 (47.7%)		11 (50.0%)	0.790
		23 (53.4%)		
		20 (46.6%)		

p-values were calculated using the Chi-Square test to study the differences between the FGFR4-388gly (Gly) and FGFR4-388Arg (Arg) variants expressing patient subgroups.

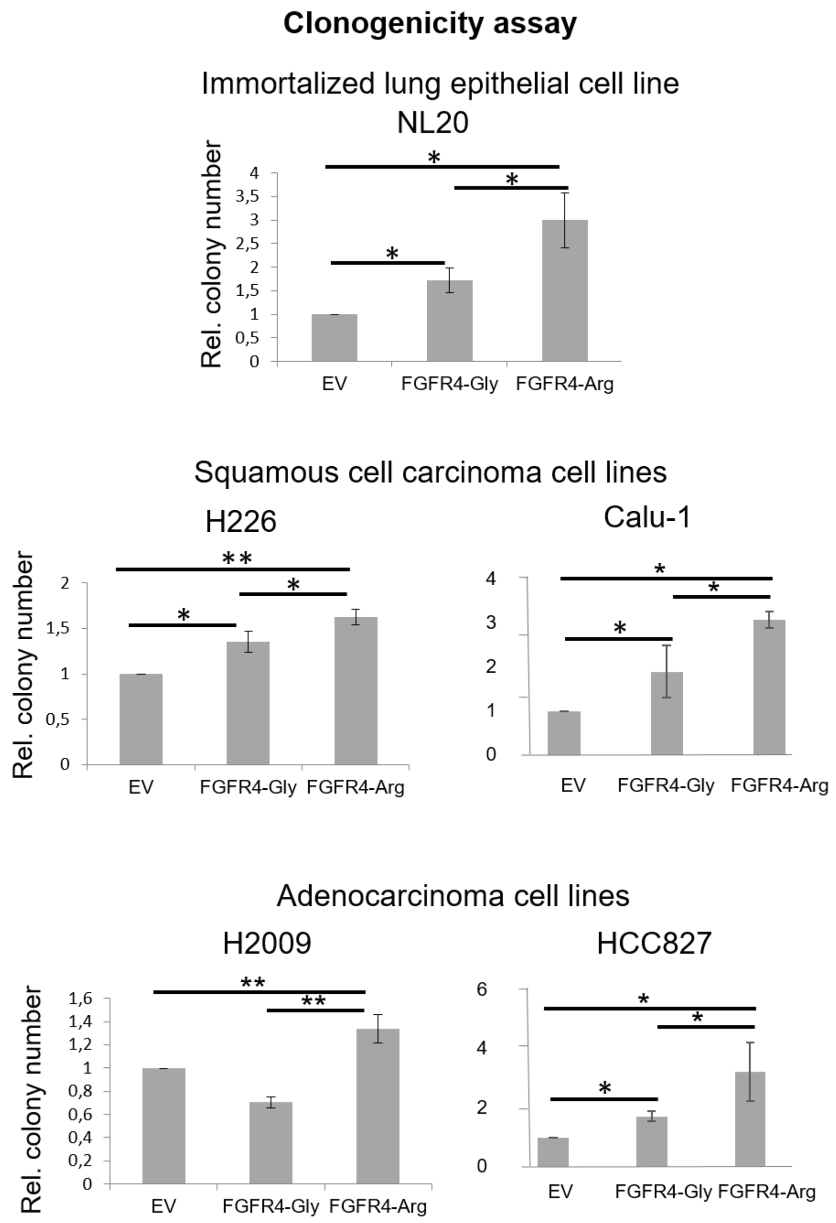

Supplementary Figure S1. Clonogenicity assays for FGFR4-388Gly- and FGFR4-388Arg-overexpressing NL20, H226, Calu-1, H2009 and HCC827 cells. The value for each replicate was normalized to that for the empty vector control in each experiment, and the mean of all normalized replicates is presented. P values (Student´s t-test) are indicated by asterisks (*p<0.05; **p<0.01; ***p<0.001). EV=Empty vector control, FGFR4-Gly=FGFR4-388Gly overexpressing, FGFR4-Arg=FGFR4-388Arg overexpressing, FBS=fetal bovine serum.

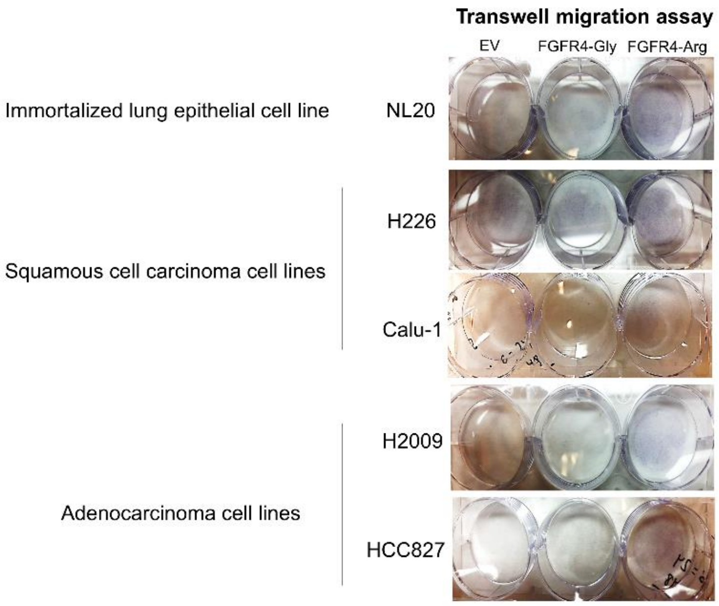

Supplementary Figure S2. FGFR4-388Arg overexpression increases migration in NSCLC cell lines. Representative images of the wells after the migration assay exhibiting the purple stained migrated cells, fixed in the bottom of the well. EV=Empty vector control, FGFR4-Gly=FGFR4-388Gly overexpressing, FGFR4-Arg=FGFR4-388Arg overexpressing, I=immortalized, SCC=Squamous cell carcinoma, ADC=Adenocarcinoma.
